# Exploring the Inhibitory Potential of Phytosterols β-Sitosterol, Stigmasterol, and Campesterol on 5-Alpha Reductase Activity in the Human Prostate: An In Vitro and In Silico Approach

**DOI:** 10.3390/plants13223146

**Published:** 2024-11-08

**Authors:** Mădălina-Georgiana Buț, Amelia Tero-Vescan, Amalia Pușcaș, George Jîtcă, Gabriel Marc

**Affiliations:** 1 Doctoral School of Medicine and Pharmacy, I.O.S.U.D., George Emil Palade University of Medicine, Pharmacy, Science and Technology of Târgu Mures, 540139 Târgu Mureș, Romania; madalina.but@umfst.ro; 2 Department of Biochemistry, Faculty of Pharmacy, George Emil Palade University of Medicine, Pharmacy, Science and Technology of Târgu Mures, 540139 Târgu Mureș, Romania; amalia.puscas@umfst.ro; 3 Department of Pharmacology and Clinical Pharmacy, Faculty of Pharmacy, George Emil Palade University of Medicine, Pharmacy, Science and Technology of Târgu Mures, 540139 Târgu Mureș, Romania; george.jitca@umfst.ro; 4 Department of Pharmaceutical Chemistry, Iuliu Hațieganu University of Medicine and Pharmacy, 41 Victor Babeș Street, 400012 Cluj-Napoca, Romania; marc.gabriel@umfcluj.ro

**Keywords:** phytosterols, steroidal 5α-reductase type 2, benign prostatic hyperplasia, testosterone, dihydrotestosterone

## Abstract

Steroidal 5α-reductase type 2 (S5αR2) is a key enzyme involved in the conversion of testosterone (TST) to dihydrotestosterone (DHT), a crucial process in the development of benign prostatic hyperplasia (BPH). Phytosterols (PSs), natural plant-derived compounds, have been proposed as potential inhibitors of S5αR2, but studies on their efficacy are limited. This study evaluates the inhibitory effects of three PSs (β-sitosterol, stigmasterol, and campesterol) on S5αR2 activity using a combined in vitro and in silico approach. The inhibitory activity of the respective PSs was assessed in vitro, by measuring TST and DHT, while molecular docking and dynamics explored PS interactions with S5αR2’s active site. The in vitro tests indicated significantly higher IC_50_ values (β-sitosterol, 3.24 ± 0.32 µM; stigmasterol, 31.89 ± 4.26 µM; and campesterol, 15.75 ± 5.56 µM) for PSs compared to dutasteride (4.88 × 10^−3^ ± 0.33 µM), suggesting a lower efficiency in inhibiting S5αR2. The in silico studies confirmed these observations, explained by the lower binding affinity identified for PSs to the enzyme’s active site in the molecular docking studies and the reduced stability of the interactions with the active site of the enzyme during the molecular dynamics simulations compared to dutasteride. The results suggest that PSs exhibit low-to-negligible inhibitory activity against S5αR2 (µM range) compared to the synthetic inhibitor dutasteride (nM range). Among the three PSs studied, β-sitosterol showed the highest inhibitory activity and the best stability in its interaction with S5αR2, when compared with stigmasterol and campesterol.

## 1. Introduction

Androgen-dependent processes, such as the regulation of urinary flow in men, male sexual differentiation during fetal development, the development and maintenance of male secondary sexual characteristics, spermatogenesis, the stimulation of anabolism, and the regulation of libido, are mediated through androgen receptors (ARs) [1]. ARs are activated by binding to endogenous agonists, which in turn leads to the modulation of specific gene expression at the nuclear level [2]. The activity of these processes is closely linked to the action of steroidal 5-alpha reductase type 2 (S5αR2), an enzyme which catalyzes the conversion of testosterone (TST) into dihydrotestosterone (DHT), a metabolite with a much higher affinity for the AR, essential for amplifying androgenic signaling in these critical processes. DHT primarily affects key areas such as the following: the prostate, where it plays a significant role in the development and progression of benign prostatic hyperplasia (BPH); hair follicles, contributing to male pattern baldness; sebaceous glands, influencing conditions like acne; and male genitalia, where it is crucial for development and sexual function. These areas are particularly sensitive to the androgenic effects mediated by DHT [3,4].

**Figure 1:**
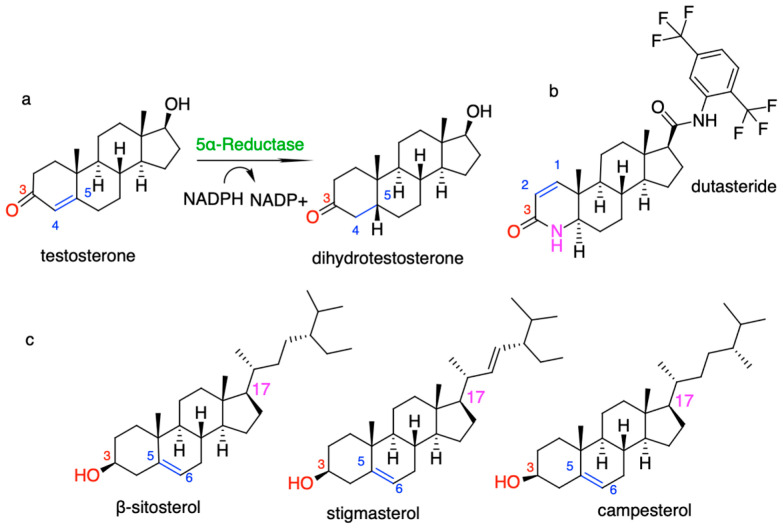
(**a**) Conversion of testosterone (TST) to dihydrotestosterone (DHT) catalyzed by S5αR2; (**b**) structure of the synthetic inhibitor (dutasteride); and (**c**) structures of natural inhibitors (β-sitosterol, stigmasterol, and campesterol). All the structures highlight the functional groups involved in interactions with S5αR2 (the hydroxyl group at position 3 in the structure of the PS, as well as the ketone group in the structure of TST, DHT, or DUT, are depicted in red; the double bond is depicted in blue; the amino group of DUT is depicted in pink), and all three PSs have different substituents attached at the C_17_ position of the sterane nucleus.

S5αR2 is part of the NADPH-dependent oxidoreductase class and is a transmembrane enzyme that includes seven α-helix transmembrane domains interconnected by six flexible loops that contribute to the structure of the enzyme’s internal cavity. The N-terminal group is oriented towards the lumen of the endoplasmic reticulum, while the C-terminal group is oriented toward the cytosol. Within the enzyme’s active site, tyrosine (Tyr) and glutamic acid (Glu) sidechains facilitate catalysis by forming hydrogen bonds with the ketone group of the substrate, promoting hydrogen transfer and the formation of an enolic intermediate, which is subsequently protonated, resulting in a saturated ketone product and NADP^+^ (Figure 1a). Regarding the inhibition mechanism, synthetic inhibitors such as finasteride and dutasteride act as irreversible competitive inhibitors, forming a covalent NADP–dutasteride/finasteride adduct in the enzyme’s active site, which explains their strong and irreversible action on S5αR2 [5].

Although the exact cause of BPH is not fully understood, it is known that a central role is played by the growth of stromal and epithelial cells caused by an excess of androgens [6]. One of the key mechanisms involved in BPH development is the conversion of TST to DHT, a more active metabolite, mediated by S5αR2. A therapeutic approach to managing BPH involves inhibiting the activity of S5αR2, and synthetic inhibitors are commonly used for this purpose [7]. Therefore, in the international guidelines for BPH treatment, three main classes of drugs are established: alpha-blockers (such as prazosin, tamsulosin, doxazosin, and alfuzosin), phosphodiesterase-5 inhibitors (only tadalafil is approved by the FDA), and S5αR2 inhibitors (only dutasteride and finasteride are approved) [8,9,10]. However, some patients with BPH do not respond adequately to these FDA-approved treatments and may experience sexual dysfunction, significantly affecting their quality of life [11].

To address the limitations of these treatments, researchers are exploring alternatives, such as plant-based inhibitors of S5αR2. Phytosterols (PSs), natural compounds derived from plants, have garnered attention due to their anti-inflammatory, antioxidant, and pro-apoptotic properties, which enable them to reduce inflammation, neutralize free radicals, and induce tumor cell death [12,13]. Additionally, few studies suggest that these compounds can inhibit S5αR2, thereby reducing the DHT levels, making them potential therapeutic agents for androgen-dependent conditions such as BPH [13]. Specifically, PSs, such as β-sitosterol, stigmasterol, and campesterol, are being promoted as promising therapeutic options for alleviating BPH symptoms. These compounds share structural similarity with the endogenous substrate (TST) (Figure 1a) and synthetic inhibitors such as finasteride or dutasteride (Figure 1b), including a sterane core structure (the base skeleton of steroids), a hydroxyl group at C_3_, and a double bond at C_5_-C_6_ of the core skeleton, which are essential for inhibiting the catalytic process (Figure 1c); for a review, see [14].

Recently, numerous plant-based dietary supplements have entered the market, claiming to inhibit the activity of S5αR2 and alleviate symptoms associated with BPH. However, many of these dietary supplements often lack robust scientific evidence to support their efficacy and safety, raising questions about their actual benefits. Many of these formulations incorporate blends of botanical ingredients such as saw palmetto, nettle root, or pumpkin seed oil, marketed as natural substitutes for pharmaceutical S5αR2 inhibitors [13].

The literature is limited in studies that specifically evaluate the potential of PSs to inhibit S5αR2 through in vitro experiments, though some suggest a possible inhibitory effect. This highlights the need for further research to clarify the underlying mechanisms and determine the therapeutic applicability of these compounds in terms of their anti-androgenic mechanisms. Moreover, there are no studies providing a detailed understanding of how PSs interact at the molecular level with S5αR2.

This study aims to investigate the inhibitory effects of PSs on S5αR2 using both in vitro and in silico methods. The in vitro methods allow the evaluation of the inhibitory activity of PSs on S5αR2 isolated from human prostate tissue. Additionally, in silico studies, which include molecular docking and molecular dynamics, provide a detailed perspective on the molecular interactions between PSs and S5αR2, thus contributing to the elucidation of the mechanisms through which these compounds act. Through this integrated approach, this study seeks to assess the inhibitory potential of PSs and provide a deeper understanding of the stability and efficacy of their interactions with S5αR2. This information could contribute to the development of more effective and safer natural therapeutic strategies for BPH management.

## 2. Results

### 2.1. Optimization of the In Vitro Enzymatic Reaction

Based on these experiments, optimal experimental conditions were established as follows: TST concentration, 20 ng/mL; protein equivalent (S5αR2) concentration, 44.69 µg/mL; NADPH concentration, 150 µM; and incubation time, 60 min at 37 °C. The extraction yields were 80.85 ± 3.95% for TST and 80.53 ± 15.57% for DHT (N = 5 replicates).

**Figure 2:**
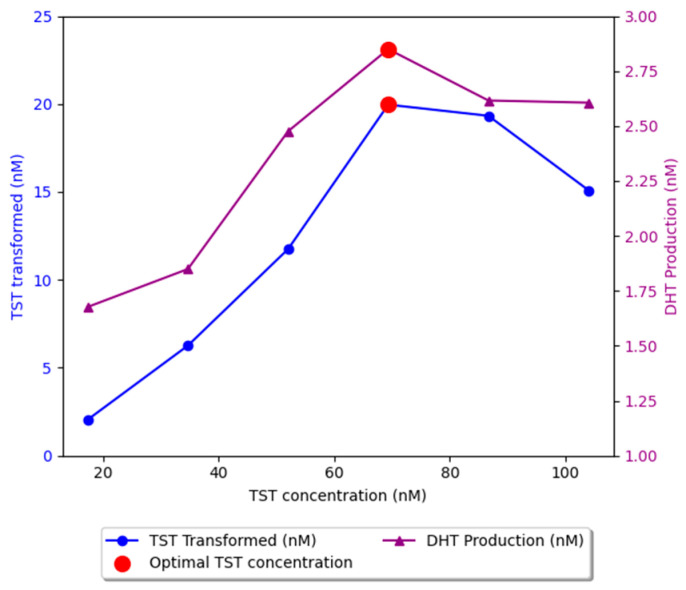
Evaluation of S5αR_2_ activity based on substrate (TST) concentration.

Figure 2 shows that, at low TST concentrations, the S5αR2 is undersaturated, meaning that not all active sites are occupied. At this stage, the reaction rate is proportional to the substrate concentration, as more substrate molecules can bind to the enzyme (concentration 5–20 ng/mL). As the TST concentration increases, more enzyme active sites become occupied, which simultaneously leads to an increase in the concentration of the reaction product (DHT). At a certain point, all active sites are occupied, and the S5αR2 is likely saturated (>20–30 ng/mL). As a result, 20 ng/mL is the optimal TST concentration to achieve the highest conversion rate to DHT, indicating the maximum efficacy of S5αR2 under these conditions.

**Figure 3:**
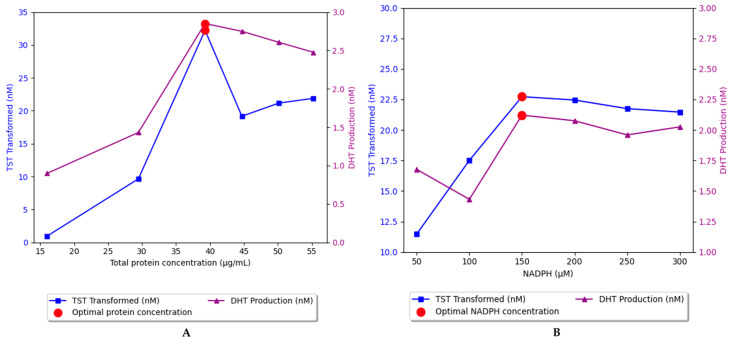
Evaluation of S5αR2 activity based on enzyme (**A**) and cofactor (**B**) concentrations.

Regarding the influence of S5αR2 concentration (reported as total protein concentration), as *the protein concentration increased*, both the amount of TST consumed and DHT production increased simultaneously. At *lower protein concentrations*, the reaction rate increased proportionally with the S5αR2 concentration, as more enzyme molecules became available to catalyze the conversion of the substrate into the reaction product. However, at concentrations greater than 45 µg/mL, the amount of TST consumed remained constant. Very high enzyme concentrations can lead to protein aggregation or other stability issues, such as increased viscosity, which may hinder enzymatic reaction efficiency and reduce activity (Figure 3A). In some cases, the reaction product may act as an enzyme inhibitor. Thus, the rapid accumulation of the product, caused by excessive enzyme concentrations, can lead to the inhibition of enzyme activity, negatively impacting the overall efficiency of the reaction [15].

At low concentrations of NADPH, the amount of TST consumed was minimal, thereby confirming the relationship between NADPH and S5αR2. The amount of TST consumed increased with increasing concentrations of NADPH, and, at concentrations above 150 µg/mL, it remained constant. Since concentrations above approximately 100 µg/mL are considered supraphysiological [16,17] and considering that NADPH is a costly chemical, a concentration of 150 µg/mL NADPH was used for the enzymatic reaction, which resulted in a satisfactory reaction rate.

### 2.2. Enzyme Inhibitor Assay

**Figure 4:**
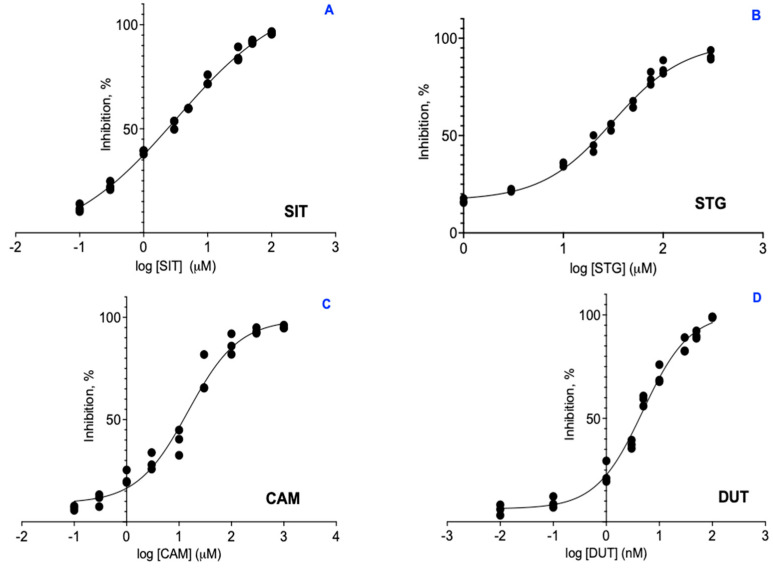
Graphical representation of IC_50_ for β-sitosterol (**A**), stigmasterol (**B**), campesterol (**C**), and dutasteride (**D**). SIT, β-sitosterol; CAM, campesterol; STG, stigmasterol; and DUT, dutasteride.

All three compounds demonstrated inhibitory activity against S5αR2, with the following IC_50_ values—β-sitosterol, 3.24 *±* 0.32 µM; stigmasterol, 31.89 *±* 4.26 µM; and campesterol, 15.75 *±* 5.56 µM—while dutasteride’s was 4.88 $\times \;10^{-3}$ ± 0.33 µM. The difference in potency between the PSs and dutasteride was significant, with the PSs having IC_50_ values in the µM range, while dutasteride had an IC_50_ value in the nM range (Figure 4).

### 2.3. Molecular Docking and Dynamics

**Figure 5:**
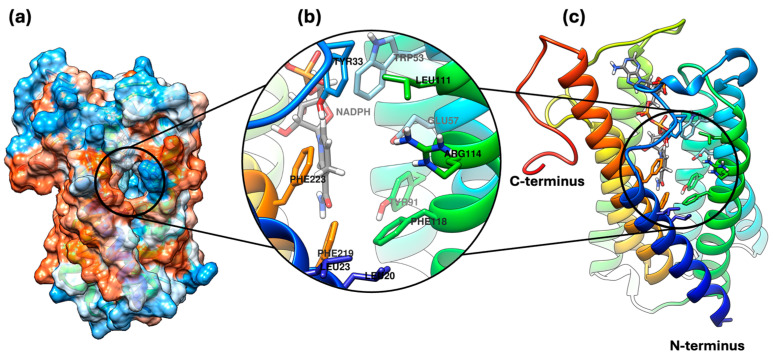
The 3D structure of S5αR2. (**a**) provides an overview of the surface of S5αR2, highlighting the potential surface interactions and areas of accessibility to the active site, which is marked with a black circle. (**b**) offers a view of the enzyme’s active site. Here, important amino acids that interact directly with the cofactor NADPH and likely with the substrate (TST) are visible. (**c**) presents the tertiary structure of the S5αR2, highlighting the helical (α-helix) structure and the locations of the N- and C-terminals. The helical structure is colored to show the orientation and folding of the protein, with a special emphasis on the active site region.

The 3D structure of S5αR2 is illustrated in Figure 5. 

The studied compounds—β-sitosterol, stigmasterol, and campesterol—along with dutasteride, the reference compound, were docked into the catalytic site of S5αR2, and the computed affinities are presented in Table 1, as variations in Gibbs’ free energy.

**Figure 6:**
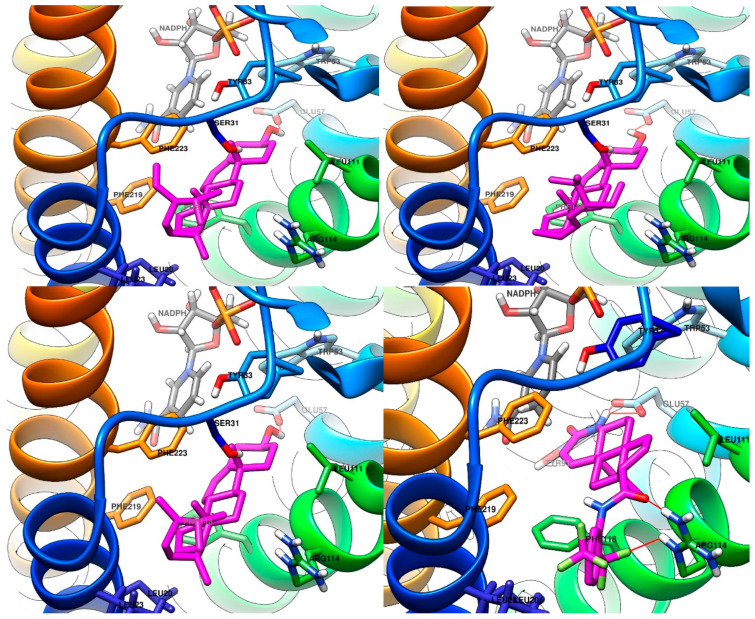
Graphical depictions of the interactions between S5αR2 and β-sitosterol (**top left**), stigmasterol (**top right**), campesterol (**bottom left**), and dutasteride (**bottom right**). The carbon atoms of the sidechains of amino acid are depicted in the corresponding color of the tertiary structure where they are found. All visible nitrogen atoms are depicted in blue; oxygen atoms are depicted in red; hydrogen atoms are depicted in white; and fluorine atoms are depicted in green. The carbon atoms of NADPH are depicted in gray, and the carbon atoms of the ligands are depicted in magenta. All non-polar hydrogen atoms are not depicted, except for NADPH.

The graphical depictions of the predicted interactions between ligands and protein are presented in Figure 6.

**Figure 7:**
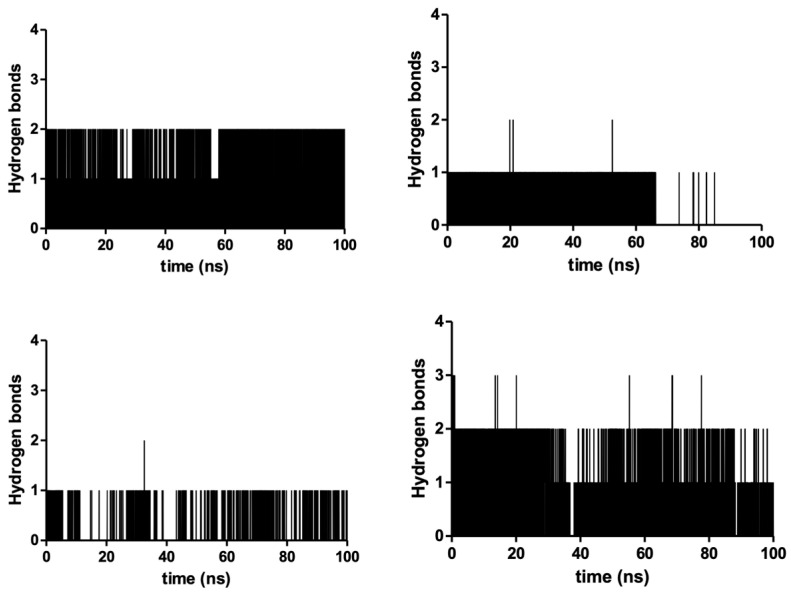
The number of hydrogen bonds between steroid S5αR_2_ and β-sitosterol (**top left**), stigmasterol (**top right**), campesterol (**bottom left**), and dutasteride (**bottom right**).

**Figure 8:**
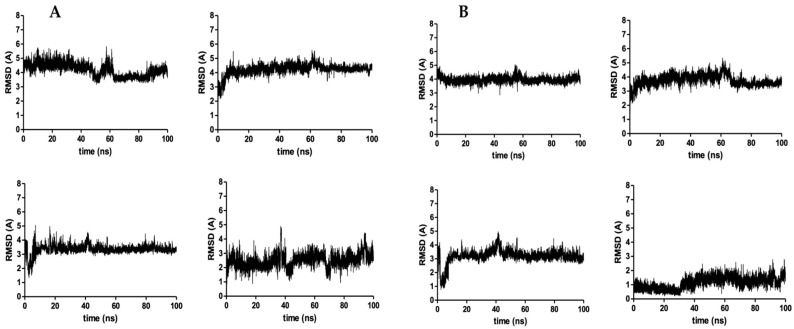
(**A**) The RMSD of β-sitosterol (**top left**), stigmasterol (**top right**), campesterol (**bottom left**), and dutasteride (**bottom right**) in the catalytic site of S5αR2. (**B**) The RMSD of the sterane of β-sitosterol (**top left**), stigmasterol (**top right**), campesterol (**bottom left**), and dutasteride (**bottom right**) in the catalytic site of S5αR2.

**Figure 9:**
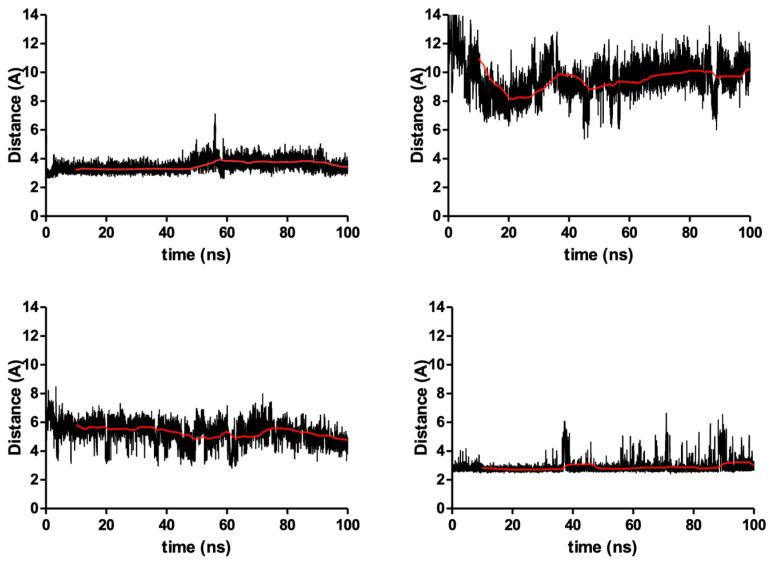
The distance between the center of mass of the phenol oxygen atom of Tyr91 from the catalytic site of S5αR2 and the oxygen alcohol from the three PSs and the oxygen atom from the cyclic amide of dutasteride, respectively: beta-β-sitosterol (**top left**), stigmasterol (**top right**), campesterol (**bottom left**), and dutasteride (**bottom right**) are all depicted in black, while in red is the 10 ns moving average.

Using the docked poses of the four compounds, their corresponding complexes with S5αR2 were subjected to a molecular dynamics study for 100 ns to evaluate their behavior in time. The main objective of the molecular dynamics study was to evaluate the stability of the interactions between the ligands and the enzyme and offer information regarding the inhibitory potential of the compounds. The results of the molecular dynamics study are presented in Table 2, and the graphical depictions are presented as follow: the number of hydrogen bonds between compounds and enzymes in Figure 7, the root mean square deviation (RMSD) of the whole ligand (Å) in Figure 8A, the RMSD of the sterane of each compound RMSD (Å) in Figure 8B, and the root mean square fluctuation (RMSF) of S5αR2’s key amino acids in Figure 9.

Taking into account the importance of Tyr91 as a key amino acid in the catalytic process and the potential inhibition of the S5αR2, we measured the distance between the center of mass of the phenol oxygen atom from Tyr91 and the oxygen alcohol from the three PSs and the oxygen atom from the cyclic amide of dutasteride, respectively. The distance between the two oxygen atoms (of each ligand and enzyme) over the 100 ns of the molecular dynamics simulations is plotted in Figure 9. The average distance between the phenol oxygen atom of Tyr91 and the ligands is presented as an average in Table 3, together with the energy contribution of Tyr91 in binding the studied ligands.

## 3. Discussion

PSs, such as β-sitosterol, stigmasterol, and campesterol, are natural compounds found in various medicinal plants. These compounds are recognized as safe and promising therapeutic options, commonly used to alleviate symptoms associated with BPH. One of the proposed mechanisms by which PSs may act in the treatment of BPH is through the inhibition of S5αR2.

The in vitro results demonstrate a significant difference in potency between PSs and dutasteride, as indicated by the IC_50_ values of PSs (β-sitosterol, 3.24 ± 0.32 µM; stigmasterol, 31.89 ± 4.26; and campesterol, 15.75 ± 5.56 µM) and dutasteride (4.88 $\times \;10^{-3}$ ± 0.33 µM). This suggests that, while PSs exhibit inhibitory activity against S5αR2, their potency is considerably lower compared to dutasteride. These findings are consistent with previously reported data in the literature. For example, Cabeza et al. (2003) demonstrated that β-sitosterol inhibits S5αR_2_ activity but with a relatively low potency, showing an IC_50_ of 2.7 µM, compared to finasteride, which has an IC_50_ of 10.12 nM [18]. Another study by Raynaud et al. (2002) reported that β-sitosterol, a PS present in the lipid sterolic extract of *Serenoa repens*, has a much lower potency as an inhibitor of S5αR2, with an IC_50_ exceeding 241.13 µM. In contrast, the free fatty acids present in the same extract, such as lauric acid and myristic acid, demonstrated a significant inhibition of S5αR2, with considerably lower IC_50_ values. Therefore, the overall inhibitory effect of the *Serenoa repens* lipid extract on S5αR2 is primarily attributed to the free fatty acids, while the contribution of β-sitosterol to this effect is relatively insignificant [19]. The difference in potency observed between the current study, the study by Cabeza et al. (2003) [18], and that by Raynaud et al. (2002) [19] could be explained by different biological contexts. Raynaud et al.’s study used an expression system in an artificial environment (Sf9), which, while useful for producing large amounts of enzyme and allowing detailed studies, may not fully reflect the conditions and behavior of enzymes in the complex and specific environment of prostate tissue. In contrast, the current study and Cabeza et al.’s study were conducted on rat prostate and human prostate cells, respectively, providing a context closer to the real conditions in the body, capturing the complex interactions between enzymes and their natural environment.

In the case of stigmasterol, a study investigated the inhibitory effects of glucosidic stigmasterol isolated from the plant *Phyllanthus urinaria* on the S5αR2 enzyme. The results showed that glycosidic stigmasterol exhibits inhibitory activity against S5αR2, with an IC_50_ value of 27.2 µM, similar to the one obtained in our study (31.89 ± 4.26 µM) [20]. No information was found in the literature regarding the potency of campesterol in inhibiting S5αR2 activity. To explore in more detail the interactions between PSs and S5αR_2_, an in silico evaluation of the ligand–S5αR2 interactions using molecular docking and molecular dynamics was proposed.

An analysis of the results of molecular docking indicates a considerable difference in terms of binding affinity between the three PSs studied—β-sitosterol, stigmasterol, and campesterol—when compared with dutasteride, used as a reference compound. Dutasteride exhibited the highest affinity for the catalytic site of the enzyme (ΔG = −10.4 kcal/mol), while the three PSs were predicted to have lower ones (stigmasterol, ΔG = −9.6 kcal/mol; β-sitosterol, ΔG = −9.5 kcal/mol; and the lowest one for campesterol, ΔG = −9.2 kcal/mol). The resulting data from the molecular docking study suggest a significant difference in terms of affinity observed between the three studied PSs and dutasteride, which could influence their inhibitory activity on S5αR2.

The visual analysis of the depiction of the best binding poses of each of the three PSs in the active site of S5αR2 indicates a very good superposition of sterane (cyclopentanoperhydrophenanthrene) for the three compounds, with small differences in the orientations being identified for the substituents on position 17 of the sterane. All three PSs are predicted to interact with the active site of the enzyme via a hydrogen bond. The alcohol moiety from the structure of the ligands is predicted to be the donor and the sidechain of Glu57 as a hydrogen bond acceptor in the respective hydrogen bonding. Other favorable interactions of the PSs with the enzyme are mainly hydrophobic ones, involving the hydrophobic sidechains of Leu20, Leu23, Ala24, Leu111, Phe118, Phe219, and Phe223. From a negative point of view for the binding of the three studied PSs, it is worth mentioning the unfavorable nearby residue of Arg114 (positively charged), which has no complementary moieties or atoms in the studied ligands, which can lead to repulsion. Moreover, the sidechain of Ser31 is likely to influence, in a negative way, the binding of the ligands (especially in terms of dynamics), due to the hydrophobic character of the sterane core found approximately 4 Å away from it.

The visual analysis of the interaction of dutasteride with the active site of S5αR2 indicates good complementarity with the enzyme. First, a hydrogen bond of the cyclic amide of dutasteride is predicted to occur with the sidechain of the key amino acid, Glu57. Second, the oxygen atom of the exocyclic amide of dutasteride and one from the trifluoromethyl group are involved in interactions with the positively charged sidechain of Arg114. Last, the sidechain of Ser31, not interacting in the pose predicted by the molecular docking study with dutasteride, is favorably oriented towards the exocyclic amide of dutasteride (3.8 Å distance). In the case of a drift of the molecule of dutasteride in dynamic conditions and the weakening of the ion–dipole bond between the sidechain of Arg114 and the amide oxygen atom, the sidechain of Ser31 would keep dutasteride in its position, thanks to the newly formed hydrogen bond.

To have a clear understanding of the interactions of β-sitosterol, stigmasterol, campesterol, and dutasteride with S5αR2 at a molecular scale and study the evolution of their complexes over time, the molecular docking study was continued with a molecular dynamics study. A large number of research papers published in recent years report in silico research, searching for potential inhibitors of S5αR, for both types 1 and 2 [21,22,23,24,25,26]. Having said this, molecular docking is known to generate false-positive results, which need to be further filtered [27,28,29,30]. One of the techniques to distinguish true-binding ligands from false-binding ligands is molecular dynamics. On top of refining molecular docking results, molecular dynamics simulations provide significant contributions to complement and enhance molecular docking studies by evaluating the behavior of the complex over time, involving water, ions, and temperature, bringing a more realistic image of the evolution over time of the bonds between ligand and protein, giving detailed mechanistic insights.

The predicted compound–enzyme complexes resulting from the molecular dynamics study were prepared for the molecular dynamics simulations, and their behavior was studied for 100 ns. Although some papers report using T = 300 K in molecular dynamics studies [21,23,25], we considered T = 310 K to be more realistic for biological human environments, as stated by other papers [5,22,24].

Hydrogen bonding between the ligand and the enzyme is an important descriptor, characterizing the stability of their complex. According to the data presented in Table 2, what stands out is the low number of hydrogen bonds between S5αR2 and campesterol or stigmasterol. The hydrogen bond of an alcohol moiety as a donor to Glu57, acting as the acceptor, appears and disappears during the simulation, indicating the instability of the interaction at hand. Significantly better average hydrogen bonding could be identified for β-sitosterol (0.73/frame) and dutasteride (0.79/frame).

Another important descriptor of the stability of a ligand–protein complex is the RMSD of the bound ligand in the active site of the enzyme. Because all four compounds share a common rigid sterane core and a flexible substituent, the RMSD for compounds was computed as a whole and for the sterane core, to prevent biasing the results by removing from calculations the movement of the flexible substituents of compounds. All three PSs presented high movement in the active site of the enzyme, characterized by a high RMSD of over 3 Å.

The visual analysis of the trajectories of the ligands during the molecular dynamics simulations indicated a high degree of flexibility of the substituents from position 17 of the ligands, which led to an artificial increase in the RMSD of the PSs, as long as the hydrocarbon substituents were not interacting with amino acid residues found nearby. Therefore, we evaluated only the movement of the sterane core of the compounds, specifically the movement of the substituent from position 17 of the sterane in all three PSs (Figure 1c). The sterane core of the PSs presents high movement inside the active site of the enzyme, with RMSD > 3 Å, indicating a high degree of instability of the S5αR2–ligand complexes. In comparison, dutasteride, used as a reference compound, had an extremely different behavior. Dutasteride, taken as a whole, had an RMSD of 2.49 Å, while, for the sterane core, the RMSD was found to be 1.14 Å. The data indicate a strong and stable binding of the sterane core of dutasteride to the active site of the enzyme, while di(trifluoromethyl)benzene retains some degree of movement. Among the PSs studied, the lowest RMSD value of the sterane core was found for campesterol (3.18 Å), while that for the sterane of dutasteride was much lower (1.14 Å), indicating that PSs have a much lower binding capacity compared to dutasteride.

The analysis of the RMSF of key amino acids from the active site of the enzyme indicates that dutasteride reduced the movement of Tyr91 the most, compared to β-sitosterol, stigmasterol, and campesterol. This is important because Tyr91 is the amino acid that starts the catalytic process of TST reduction by an acid–base catalytic group to polarize the steroid C-3 carbonyl group [5], and its blockage would drastically reduce the enzymatic activity. Regarding the influence of ligands on Glu57 and Trp53, no clear pattern could be identified for the studied compounds, but this fact is not a fundamental descriptor, especially for Glu57, which intervenes in the following steps of catalysis, not in the first one. Anyhow, campesterol has the lowest influence on the movement of Tyr91, Glu57, and Trp53. Regarding the Arg114 amino acid found outside the active site, none of the studied PSs would reduce its movement, compared to dutasteride. This is thanks to the fluorine atoms of dutasteride, which would interact with Arg114 and create a supplementary bond, better stabilizing dutasteride as an inhibitor in the active site of the enzyme.

Detailed studies regarding the interaction of the studied compounds with the key amino acid Tyr91 confirm that β-sitosterol has a better interaction with the catalytic site of S5αR2, compared to stigmasterol and campesterol. The alcohol oxygen of β-sitosterol is closer to Tyr91, compared to the other two PSs, but the interaction value is lower than the threshold of 0.5 kcal/mol. The oxygen atom from the cyclic amide of dutasteride is found, during the simulation, to be close to Tyr91’s oxygen atom, and the energy decomposition of the interaction with the respective amino acid, measuring −1.16 kcal/mol, indicates that they are found to be involved in a significant interaction.

In the in silico study of the inhibitory potential of dutasteride, we focused on the initial step of the inhibition process to demonstrate the interaction between dutasteride and two key amino acids, Glu57 and Tyr91, involved in the enzyme’s catalytic activity. This approach allowed us to compare dutasteride with three phytosterols that cannot initiate the enzyme’s catalytic process, which also means that they cannot lead to the irreversible inhibition of the enzyme due to their unfavorable structural features.

The results of the molecular dynamics simulations would indicate that β-sitosterol, stigmasterol, and campesterol have a low-to-negligible inhibitory activity against S5αR2, compared to dutasteride. Between the three PSs, β-sitosterol seems to create the most stable complex with S5αR2, compared to stigmasterol or campesterol.

## 4. Materials and Methods

### 4.1. Materials

All chemicals and reagents were of analytical-grade purity and purchased from various suppliers: ethyl acetate, ethanol, and sucrose were obtained from VWR International SAS (Fontenay-sous-Bois, France); NADPH was obtained from ROTH (Karlsruhe, Germany); and EDTA and the sodium phosphate-buffered solution were from the Chemical Company (Iasi, Romania). Testosterone and dihydrotestosterone ELISA kits were obtained from Rockland Immunochemicals (Pottstown, PA, USA). The standards, β-sitosterol, BSA, DTT, and the Bradford reagent were purchased from Thermo Fisher Scientific (Waltham, MA, USA), while stigmasterol and campesterol were obtained from Sigma-Aldrich (St. Louis, MO, USA). Ultra-pure water was obtained using a Milli-Q purification system (Merck Millipore Corporation, Burlington, MA, USA).

### 4.2. Preparation of Microsomes from Prostate Tissue (Enzymatic Source)

Prostate tissue was obtained from the Urology Clinic of the County Hospital in Târgu Mureș, with the favorable approval of the ethics committee (nr. 2280/15.03.2021). To prevent degradation and maintain enzymatic activity, the prostate tissue was placed in PBS (pH 6.5), washed, flash-frozen with liquid nitrogen immediately after tissue collection, and stored at −80 °C. Each sample was thawed only once before use to minimize any potential loss of activity. The human enzymatic source was prepared according to the procedures described below [31,32,33,34], with some modifications. The prostate tissue (200 mg), previously weighed, was cut into small pieces and homogenized (UltraTurrax T10, IKA, Staufen, Germany) with 5 mL of medium A (20 mM sodium phosphate (pH = 6.5), 0.32 mM sucrose, 0.1 mM DTT, and 1 mM EDTA). The resulting homogenate was centrifuged at 4000× *g* rpm for 15 min, and the supernatant was used as the enzymatic source [29,30,31]. All procedures were performed at 4 °C. The enzyme’s concentration in the supernatant was estimated using the Bradford protein estimation method [35].

### 4.3. Determination of 5-Alpha Reductase Enzymatic Activity

To evaluate the activity of S5αR2 in human prostate tissue, a method from the literature [29] was used with the following modifications. The enzymatic reaction mixture included 44.69 µg of microsomal protein from the prostate, 20 ng/mL TST in ethanol, test substance (β-sitosterol, stigmasterol, campesterol, and dutasteride), 1 mM DTT, and 40 mM sodium phosphate buffer (pH 6.5) in a final volume of 400 μL. The enzymatic reaction was initiated by adding 50 μM NADPH, and the reaction mixture was incubated in a shaking water bath at 37 °C for 60 min. The enzymatic reaction was terminated by adding 500 µL of ethyl acetate and homogenized with a vortex mixer for 2 min. In the next step, TST and DHT were extracted through a liquid–liquid microextraction. The organic phase was evaporated to dryness, and the residues were reconstituted with ethanol. The concentrations of TST and DHT were determined using the ELISA technique.

To establish the optimal conditions for the conversion of TST to DHT in the presence of S5αR2, different final concentrations of TST (5, 10, 15, 20, 25, and 30 ng/mL) were tested at five different protein concentration (16, 29.45, 39.27, 44.69, 50.12, and 55.23 µg protein equivalent). Additionally, various cofactor concentrations (50, 100, 150, 200, 250, and 300 µM) were also tested.

### 4.4. Enzyme Inhibitor Assay Procedure

The inhibitory activity against S5αR2 was calculated as follows:

$$\mathrm{Reaction}\;\mathrm{rate}\;\left(R,\;\%\right)=\frac{\mathrm{DHT}}{\mathrm{TST}+\mathrm{DHT}}\times 100\;\mathrm{Inhibition}\;\left(\%\right)=\left[1-\frac{\left(R_{\mathrm{sample}}-R_{\mathrm{blank}}\right)}{\left(R_{\mathrm{control}}-R_{\mathrm{blank}}\right)}\right]\times 100$$

where DHT and TST represent the concentrations of DHT and TST obtained after the enzymatic reaction. R_sample_, R_blank_, and R_control_ represent the reaction rate in the presence of test substances, the reaction rate measured in the absence of the test sample and the enzyme inhibited at time zero, and the reaction rate measured in the absence of test substances, respectively.

To determine the IC_50_ values of the inhibitors under investigation, a series of experiments were conducted in which the percentage of enzyme inhibition was measured at various concentrations of each tested inhibitor. The data were fitted to a non-linear regression model using GraphPad Prism (version 9.5) to generate a dose–response curve. The IC_50_ value, defined as the concentration of inhibitor required to achieve 50% inhibition of enzymatic activity, was automatically calculated from the curve. A range of concentrations was selected to adequately cover a wide range around the estimated IC_50_ values (Figure 4). This included dutasteride (0.1–1000 nM), sitosterol (0.1–100 μM), stigmasterol (1–300 μM), and campesterol (0.1–1000 μM). These concentration ranges provided sufficient data to plot accurate inhibition curves and determine IC_50_ values with high precision. Each experiment was performed in triplicate.

### 4.5. Molecular Docking and Dynamics Procedure

The 3D structure of S5αR2 was obtained from RCSB Protein Data Bank, with code 7BW1 [5,36]. Due to the absence of some amino acids in the crystallized structure, a 3D structure was constructed by homology modeling using SWISS-MODEL (GMQE = 0.91) [37]. The preparation of the protein to be used as a target in the molecular docking study employed AutoDock Tools 1.5.6, following the previously reported protocol [38,39,40]. The search space was centered on the coordinates x = −25.542, y = 12.397, and z = 23.048 of the modeled protein, with sides equal to 22, to include the amino acids Leu20, Leu23, Tyr33, Trp53, Glu57, Tyr91, Leu111, Arg114, Phe118, Phe219, and Phe223, involved in the bonding of substrates and inhibitors, as previously reported [5]. The molecular docking study was conducted with AutoDock Vina 1.1.2, with the exhaustiveness parameter set to 15 [38]. The files containing the structures of β-sitosterol, stigmasterol, campesterol, dutasteride, and NADPH were generated using Avogadro 1.2.0 [41], optimized using MMFF94 [42], and they were further processed using AutoDock Tools 1.5.6 to be used as ligands in the molecular docking study [39,40]. The visual results of the molecular docking study were analyzed using UCFS Chimera 1.10.2 [43].

The molecular dynamics study of the top binding pose of each ligand with S5αR2 and NADPH as a cofactor was performed using GROMACS 2023 on a computer running Debian 11 with Intel 12700KF CPU with CUDA 12 for parallelization on an NVIDIA RTX 3060 GPU [44]. The CHARMM36 force field was used, and the TIP4 water model was used for solvation of the systems. The parametrization of the ligands was carried out using CGenFF [45,46,47,48]. The salt concentration of the systems was set to 0.15 M using sodium and chloride ions, in agreement with previous reports in the field [21,22,23]. To equilibrate the systems, the NVT and NPT ensembles were employed at T = 310 K and P = 1 bar, according to reports in the literature [5,26,49]. The resulting systems, comprising approximately 40,000 atoms each, were simulated for 100 ns, and their trajectory was further analyzed. Visual inspection of the evolution of the systems was performed using VMD 1.9.4 [50]. A detailed analysis was performed for studying the interaction of the key amino acid Tyr91 and the four studied compounds in terms of distance and energy interactions, using an energy decomposition approach [51,52].

## 5. Conclusions

The present study demonstrated, by employing in vitro and in silico methodologies, that PSs such as β-sitosterol, stigmasterol, and campesterol exhibit low inhibitory activity against the S5αR_2_ enzyme, compared to synthetic inhibitors such as dutasteride. Among the three PSs analyzed, β-sitosterol appeared to form the most stable complex with S5αR_2_, both in vitro and in silico, compared to stigmasterol and campesterol.

Therefore, it can be inferred that the anti-androgenic action of PSs as S5αR_2_ inhibitors could have a relatively limited impact on their effectiveness in treating BPH. It is likely that other effects, such as anti-inflammatory, antiproliferative, and antioxidant properties or receptor antagonism, may play a much more significant role. Additionally, the claimed therapeutic benefit of plant extracts rich in PSs for BPH seems to derive more from the synergistic action of the entire plant rather than the effect of PSs against S5αR_2_ activity. Further studies are necessary to better understand the proportionate involvement of each mechanism in the overall therapeutic effect.

## Figures and Tables

**Table 1:** The results of the docking study expressed as variations in Gibbs’ free energy (kcal/mol).

Compound	ΔG
β-sitosterol	−9.5
stigmasterol	−9.6
campesterol	−9.2
dutasteride	−10.4

**Table 2:** The RMSD of the compounds as ligands and the RMSF of key amino acids of S5αR_2_ computed after the 100 ns of the molecular dynamics study (Å).

Compound	Average Compound–S5αR2 Hydrogen Bonds	Compound RMSD		S5αR2’s Key Amino Acids RMSF			
		Whole Compound	Sterane of Compound	Tyr91	Glu57	Trp53	Arg114
β-sitosterol	0.73	4.19	3.97	0.21	0.19	0.22	0.99
stigmasterol	0.32	4.22	3.75	0.22	0.64	0.21	1.01
campesterol	0.07	3.35	3.18	0.78	0.58	0.65	0.99
dutasteride	0.79	2.49	1.14	0.19	0.64	0.29	0.58

**Table 3:** The average distance between the phenol oxygen atom of Tyr91 from the catalytic site of S5αR2 and the oxygen alcohol from the three PSs and the oxygen atom from the cyclic amide of dutasteride and the energy interaction of Tyr91 over the last 25 ns of the molecular dynamics simulation.

Compound	Average Distance to Tyr91 Oxygen (±SD)	Energy Interaction with Tyr91 (kcal/mol)
β-sitosterol	3.51 (±0.45)	0.00
stigmasterol	9.57 (±1.24)	0.00
campesterol	5.30 (±0.77)	0.00
dutasteride	2.87 (±0.45)	−1.16

## Data Availability

Data are contained within the article.
